# Beam‐commissioning methodology for a three‐dimensional convolution/superposition photon dose algorithm

**DOI:** 10.1120/jacmp.v1i1.2651

**Published:** 2000-01-01

**Authors:** George Starkschall, Roy E. Steadham, Richard A. Popple, Salahuddin Ahmad, Isaac I. Rosen

**Affiliations:** ^1^ Department of Radiation Physics The University of Texas M.D. Anderson Cancer Center Houston Texas 77030‐4095

**Keywords:** photons, treatment planning, commissioning

## Abstract

Commissioning beam data for the convolution/superposition dose‐calculation algorithm used in a commercial three‐dimensional radiation treatment planning (3D RTP) system (${\text{PINNACLE}}^{3}$, ADAC Laboratories, Milpitas, CA) can be difficult and time consuming. Sixteen adjustable parameters, as well as spectral weights representing a discrete energy spectrum, must be fit to sets of central‐axis depth doses and off‐axis profiles for a large number of field sizes. This paper presents the beam‐commissioning methodology that we used to generate accurate beam models. The methodology is relatively rapid and provides physically reasonable values for beam parameters. The methodology was initiated by using vendor‐provided automodeling software to generate a single set of beam parameters that gives an approximate fit to relative dose distributions for all beams, open and wedged, in a data set. A limited number of beam parameters were adjusted by small amounts to give accurate beam models for four open‐beam field sizes and three wedged‐beam field sizes. Beam parameters for other field sizes were interpolated and validated against measured beam data. Using this methodology, a complete set of beam parameters for a single energy can be generated and validated in approximately 40 h. The resulting parameter values yielded calculated relative doses that matched measured relative doses in a water phantom to within 0.5–1.0% along the central axis and 2% along off‐axis beam profiles for field sizes from 4cm×4cm to the largest field size available. While the methodology presented is specific to the ADAC ${\text{PINNACLE}}^{3}$ treatment planning system, the approach should apply to other implementations of the dose model in other treatment planning system.

PACS number(s): 87.53.–j, 87.66.–a

## INTRODUCTION

Radiation treatment planning systems that support three‐dimensional (3D) visualization and dose computation are becoming more prevalent in radiation oncology clinics. Although users realize that 3D radiation treatment planning (3D RTP) provides increased capabilities over the more conventional two‐dimensional radiation treatment planning (2D RTP), they are discovering that the 3D RTP process and its treatment planning systems require significantly more quality assurance support.^1^ The simulation of radiation beams in 3D RTP systems is more complex than that in 2D RTP systems, relying more on beam models rather than on tabulations and modifications of measured data.^2^ ^–^ ^4^ The commissioning of a clinical treatment beam, i.e., acquiring the appropriate parameters to support the dose‐calculation model for the particular beam configuration, is a task of major significance for a model‐based dose‐calculation algorithm. It is much more difficult to acquire the appropriate parameters for a sophisticated beam model used in a 3D RTP system than for data‐driven beam models such as those commonly used in 2D RTP systems. Papers have been presented to assist the physicist in this task,^5^ ^–^ ^8^ illustrating the difficulty of the procedure.

The task of commissioning a beam model entails determining a set of beam parameters from a restricted set of beam data, ensuring that these parameters accurately fit measured data for other field sizes, and demonstrating reasonable parameter trends as functions of field size. The purpose of this paper is to describe a methodology for beam commissioning that not only fulfills these criteria, but also enables the physicist to commission a set of beam data in a reasonable amount of time.

The present work describes a methodology we applied to commissioning photon beams for one commercial 3D RTP system, the ADAC ${\text{PINNACLE}}^{3}$, v4.2f (ADAC Laboratories, Milpitas CA). Although the details of this methodology are specific to one particular system, the principles upon which it is based can be applied to the commissioning of beam parameters for other 3D RTP systems. This paper first describes the dose‐calculation algorithm and the various beam parameters used in the algorithm. The goals, methodology, and documentation of the beam‐commissioning process are then discussed. Finally, the results of commissioning photon beams for a 3D RTP system at The University of Texas M. D. Anderson Cancer Center are presented with suggestions for new or additional software to aid the physicist in the process of beam commissioning.

## METHODS

### Dose‐calculation algorithm

The photon dose‐calculation algorithm is the convolution algorithm,^9^ which was introduced by Mackie *et al.*,^10^ and extended by Papanikolaou *et al.* ^11^ to polyenergetic spectra. The dose that is absorbed at a point **r**, denoted as *D*(**r**), is expressed as the convolution of the total energy released per unit mass (terma) with a convolution kernel. This kernel represents the relative energy deposited per unit volume in the vicinity of the site of the primary photon interaction. The dose is given by the following equation:

(1) $$D(r)=\int_{V}\int_{E}dEd^{3}r^{\prime }\frac{\mu }{\rho }(r^{\prime },E)\frac{d\Psi (r^{\prime },E)}{dE}A(r-r^{\prime },E),$$

where μ/ρ is the mass attenuation coefficient, $d\Psi (r^{\prime },E)/dE$ is the energy fluence spectrum at position $A(r-r^{\prime },E)$ is the convolution kernel. To simplify the four‐dimensional integration, Papanikolaou *et al.* ^11^ separated the energy integration from the spatial integration and approximated the dose using

(2) $$D(r)=\int_{V}d^{3}rT(r^{\prime })\bar{A}(r,r-r^{\prime }),$$

where *T*(**r**′) is the terma distribution, expressed as

(3) $$T(r^{\prime })=\int_{E}dE\frac{\mu }{\rho }(r^{\prime },E)\frac{d\Psi (r^{\prime },E)}{dE},$$

and $\bar{A}(r,r-r\prime )$ is the polyenergetic dose‐spread kernel, averaged over the local spectrum of the beam. Because the energy spectrum is position dependent, the spectrally averaged kernel is also position dependent. In the present implementation of this algorithm, the photon spectrum is represented by a set of relative photon fluence values at 15–20 discrete energies. The relative spectral weights are variables that can be modified by the physicist to fit calculations to the measured dose distributions.

The photon spectrum is assumed to soften with its transverse distance from the central axis of the beam. Each spectral weight $W_{i}$ is reduced by an off‐axis factor of

(4) $${\left[\frac{1}{1+(E_{i}/E_{\max})}\right]}^{S\theta },$$

where $E_{i}$ is the energy of the *i*th spectral bin, θ is the angle that a ray line makes with the central axis, and *S* is an off‐axis softening parameter, which can be modified by the user in fitting the measured dose distributions.

The off‐axis dependence of the in‐air photon fluence incident on the patient is modeled as a cone, with the in‐air fluence increasing linearly with distance from the central axis to a maximum cone radius. Both the cone angle, expressed in terms of the increase in fluence per unit distance, and the cone radius are adjustable parameters to be fit to the measured beam profiles. Outside the field, which is limited by the collimators, the attenuation of the fluence by the collimators is modeled based on a jaw transmission factor.

Scatter from the flattening filter is modeled by adding to the in‐air photon fluence a contribution, which is calculated by convolving a Gaussian distribution that has a specified height and width with a unit mask whose dimensions are identical to that of the field as defined by the collimators.^12^

Scatter from beam modifiers such as wedges and compensating filters is modeled by modifying the fluence by a factor equal to 1+MSF×L. In this expression, *L* is the length of the primary ray through the modifier, and MSF, the modifier scatter factor, is another parameter whose value is adjusted to fit calculations to the measured wedged beam profiles. The modifier scatter factor adjusts the slope of the off‐axis profile of the wedged beam profiles.

Focal‐spot blurring is modeled by convolving the in‐air photon fluence with a Gaussian distribution function whose full‐width‐half‐maximum values in the *x* and *y* directions are represented by source‐size parameters. These parameters are adjusted to fit calculations to measured beam profiles in the penumbra of the beam.

Finally, electron contamination in the photon beam is modeled by adding to the dose an electron component that is the product of a depth dependent factor and an off‐axis factor. The electron‐dose component is given by the empirical equation

(5) $$D_{e}(r,d;fs)=F_{D}(d;fs)F_{0A}(r).$$

In Eq. (5), the depth‐dependent factor is given by

(6) $$\begin{array}{lll} F_{D}(d;fs) & = & \frac{E_{fs}(fs)}{SF}\frac{e^{-Kd}-e^{-Kd_{m}}}{1-e^{-Kd_{m}}},\;\;\;\;d>DFd_{m}\\ & = & F_{fs}(fs)\left[1+\frac{SF-e^{-KDFd_{m}}+(1-SF)e^{-Kd_{m}}}{SF(1-e^{-Kd_{m}})DFd_{m}}d\right],\;\;\;\;\;d<DFd_{m}\\ & = & 0,\;\;\;\;\;\;d>d_{m}. \end{array}$$

**Figure 1:**
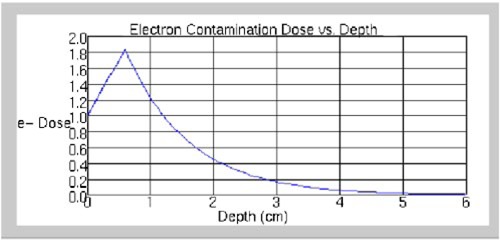
(Color) Central‐axis depth‐dose distribution for electron‐contamination component. Values of parameters are as follows: $d_{m}=6\;\text{cm},\;k=1\;{\text{cm}}^{-1},F(10\times 10)=1,(DF)=0.1,\;SF=0.3$.

Qualitatively, this expression is an exponential decreasing to 0 at $d_{m}$, the maximum depth of electron contamination (Fig. 1). In Eq. (6), the parameter *K* represents the steepness of the exponential, while $F_{fs}(fs)$ is the actual electron contamination at the surface. If the exponential were continued to the surface, the dose at the surface would be greater than $F_{fs}(fs)$ by a factor denoted as 1/*SF*, where SF≤1. However, at a depth between the surface and $d_{m}$, denoted as *DF* $d_{m}$ ((DF) is “depth fraction”), the electron contamination becomes linear, with a surface value of $F_{fs}(fs)$. The off‐axis factor is given by

(7) $$F_{\text{OA}}(r)=e^{-A\theta^{2}},$$

where *A* is an adjustable parameter and θ is the angle defined by the central axis and the ray line passing through the point at off‐axis distance *r*. Finally, the field‐size dependent surface dose $F_{fs}(fs)$ is given by the expression

(8) $$F_{fs}(fs)=F(10\times 10)+C_{1}(fs-10)+C_{2}(e^{-C_{3}10}-e^{-C_{3}fs}),$$

where F(10×10) is the surface dose for a 10cm×10cm field, and $C_{1}$, $C_{2}$, and $C_{3}$ are empirical parameters that are also adjustable.

### Beam‐commissioning methodology

#### Commissioning goals

Our beam‐commissioning goal was to generate photon beam models that reproduced within the limits of certain criteria measured central‐axis depth doses and off‐axis profiles over a wide range of field sizes and wedges. The criteria were the following:

(1) Central‐axis depth doses reproduced to within 0.5% of the maximum value for depths between $d_{\max}$ and 20 cm.
(2) Central‐axis depth doses reproduced to within 1.0% of the maximum value for depths greater than 20 cm.
(3) Central‐axis depth doses reproduced to within 5% of the maximum value for depths less than $d_{\max}$.
(4) Off‐axis profiles in the low‐dose‐gradient region within the beam reproduced to within 2.0% of the central‐axis value for depths less than 30 cm.
(5) Off‐axis profiles in the low‐dose‐gradient region within the beam reproduced to within 5.0% of the central‐axis value for depths greater than 30 cm.
(6) Off‐axis profiles in the high‐dose‐gradient region (penumbra) reproduced to within 2 mm of measured values.

It should be noted that these values are as good as, or better than, values indicated in the American Association of Physicists in Medicine Radiation Therapy Committee Task Group 53 report on quality assurance of treatment planning.^1^

#### Measured beam data

The measured beam data used in the commissioning process consist of a set of measured central‐axis depth doses and off‐axis profiles obtained at several depths for a large number of square fields. This data set included field sizes from 4 cm × 4 cm to the largest field size available on the treatment machine. In our institution, these beam data are acquired when accelerators are commissioned and are stored as ASCII files using a custom format. The same data are used for beam commissioning in the treatment planning system. Beam data was converted into the appropriate ASCII format for the treatment planning system. The central‐axis depth doses were normalized to give a value of 100.00% at the depth of maximum central axis dose $\left(d_{\max}\right)$. Each off‐axis profile was normalized to a value of 1.00 on the central axis.

The calculated central‐axis depth doses were normalized so that they matched the measured central‐axis depth doses at a user‐defined depth, recommended by the vendor to be a depth beyond that of electron contamination. Our calculated central‐axis depth doses are normalized to match the measured doses at 10‐cm depth. The calculated off‐axis profiles are normalized so they match the measured off‐axis profiles at the central axis. A limited number of square field sizes were used in modeling and then interpolation of the calculations was tested against the intermediate field sizes not used in modeling.

Finally, the calculated dose rates were related to measured dose rates in order to calculate monitor units. To establish these relationships, the dose per monitor unit at the normalization depth of 10 cm was obtained for each field size. These values were obtained from output factors and tissue‐maximum ratio (TMR) values previously measured for the linear accelerator when the machine was commissioned.

#### Beam parameters

The goal of beam commissioning is to determine a set of values for the adjustable parameters that generate calculated dose distributions so that these dose distributions approximate measured beam data to within a specified tolerance. Approximately 30 adjustable parameters must be determined in the commissioning process to fit the measured beam data, although not all parameters are independent. These parameters are summarized as follows.

(1) a discrete energy spectrum consisting of a set of energies and corresponding relative photon fluences, which are described by relative weights at each energy;
(2) a factor, *S*, that models off‐axis beam softening [Eq. (4)];
(3) a cone angle and a cone radius for modeling off‐axis changes in the in‐air photon fluence;
(4) a transmission factor for photon fluence through the collimators;
(5) the height and width of the Gaussian distribution of scatter from the flattening filter;
(6) a measure, MSF, of the scatter from the beam modifiers;
(7) the dimensions of the photon source; and
(8) a set of parameters that model the electron contamination:
  (a) $d_{m}$, a maximum depth of electron contamination [Eq. (6)],
  (b) *K*, a factor that describes steepness of the exponential depth dose of electron contamination [Eq. (6)],
  (c) *SF*, a factor modifying the surface dose [Eq. (6)],
  (d) *DF*, a depth at which the electron contamination curve becomes linear [Eq. (6)],
  (e) *A*, a factor that measures the rapidity at which the off‐axis component of the electron contamination goes to zero [Eq. (7)],
  (f) $C_{1}$, $C_{2}$, and $C_{3}$, parameters that alter the field‐size dependence of the electron contamination [Eq. (8)],

Before starting beam modeling, the physicist must understand how each model parameter affects the dose distribution, and the magnitude of the effect that changing each parameter has on the dose distribution. The energy spectrum, for example, primarily affects the central‐axis depth dose profile. Because the calculated depth doses are normalized to fit the measured doses at a fixed depth, usually taken to be 10 cm, making the beam harder, i.e., increasing the relative weights of the high‐energy components, increases the dose at large depths and decreases the dose at shallow depths. Softening the beam has the opposite effect on the central‐axis dose profile, decreasing the dose at largest depths and increasing it at shallow depths. Moreover, because the normalization occurs at 10 cm depth, only the highest energy components of the spectrum appear to have an observable effect on the depth dose profiles. Thus, if the calculated depth doses are larger than the measured values at large depths, one should soften the beam by reducing the spectral weights of only the two or three highest energy components. On the other hand, if the calculated doses are smaller than the measured doses at large depths, the weights of the two or three highest energy components should be increased. Typically, the spectral weights of the highest energy components range from 0 to approximately 25% of the maximum spectral weight values. The central‐axis depth dose is very sensitive to these weights, especially that of the highest‐energy component; changing this weight by as little as 0.001 has a noticeable effect on the depth dose.

Four parameters can be varied to fit the calculated off‐axis profiles to the measured values, the cone angle, the cone radius, the off‐axis softening parameter, and the beam modifier scatter parameter. The cone angle is adjusted to give good off‐axis fits in the region around the central axis. The off‐axis profile is sensitive to the cone angle, so small changes in the cone angle have significant effects on the dose away from central axis. Typical values of the cone angle range from 0.00 to $0.02\;{\text{cm}}^{-1}$. Once a good fit is achieved near the central axis, the cone radius is adjusted to fit the off‐axis profile far from the central axis. It should be noted, however, that for some machines, modeling the off‐axis profile by a cone model did not work as well as for other machines. In such cases it was more difficult to determine a suitable set of off‐axis parameters that generated calculated profiles that matched measured profiles to within acceptable tolerances. The off‐axis softening parameter controls the difference between the calculated and the measured off‐axis profiles far from the central axis at shallower depths. Increasing the off‐axis softening increases the calculated doses at large distances from the central axis, but only at shallower depths. Thus it is important to first fit the off‐axis profiles to the measured values at large depths (13–25 cm) using the cone angle and cone radius parameters, and then fine‐tune the fit at shallower depths (<13 cm) using the off‐axis softening parameter. Finally, the beam modifier scatter parameter adjusts the angle of the off‐axis profile for wedged fields. Increasing the scatter parameter decreases the dose at the thin end of the wedge and increases the dose at the thick end. Decreasing the scatter parameter increases the dose at the thin end of the wedge and decreases the dose at the thick end.

The Gaussian height, Gaussian width, and jaw transmission parameters affect the dose in the low‐dose regions of the off‐axis profiles. Jaw transmission is used to match the calculated to the measured off‐axis profiles at the largest off‐axis distance; Gaussian height and Gaussian width can be used to adjust the slope of the low‐dose region near the beam edge. The magnitude of the calculated doses in the low‐dose region is very sensitive to the value of the jaw transmission parameter. A parameter value between 0.00 and 0.06 is usually all that is necessary to obtain a good fit between the calculated and the measured doses at the largest off‐axis distance. The Gaussian height parameter should also be small (typically less than 0.05) because large values adversely affect the monitor‐unit calculations, as will be discussed in the Results. In general, larger values of the Gaussian height parameter are needed to more accurately fit the measured profiles. It may be necessary to compromise the fit of the slope of the off‐axis profile in the low dose region for the monitor units to be calculated correctly.

Finally, the electron‐contamination parameters affect the central‐axis depth dose at shallow depths $\left(\text{typically}<d_{\max}\right)$. Behavior of the electron contamination contribution was very difficult to control in the modeling process. Consequently, modeling of the energy spectrum should match the calculated to the measured central‐axis depth doses at shallow depths, resulting in a negligible contribution from the electron contamination.

While it is possible to fit a different set of the beam parameters to each set of the measured beam data, one set per field size, there is no guarantee that the beam parameters would be physically meaningful and form a self‐consistent set. Proper extrapolation to geometries with rectangular fields, blocked fields, varying skin surfaces, and internal heterogeneities requires parameter values that correctly model the physical processes. It is more desirable to determine the beam parameters from a limited set of measured data and then test the results to ensure that the models accurately reproduce the measured data for other field sizes. In commissioning a beam model, it is important to ensure that the physics is correctly incorporated in the parameter values.

The ADAC ${\text{PINNACLE}}^{3}$ treatment planning system provides automodeling software to assist in beam commissioning. This software calculates values of beam parameters based on minimizing the root‐mean‐square (RMS) difference between the measured and the calculated dose profiles in specific regions of the beam. For example, one automodeling tool determines the Gaussian height, Gaussian width, and jaw transmission parameters by minimizing the RMS difference between the measured and the calculated doses in the tails (low‐dose regions) of the off‐axis profiles. Care must be taken in applying the automodeling tools, however. The present version of these tools does not allow the user to differentially weight the profiles according to their importance. A profile at a depth of 35 cm has the same importance as a profile at a depth of 10 cm, when, in fact, the accuracy of the calculated profile at a depth of 10 cm is more crucial. For most cases, it is more important that the beam model accurately reproduce the tails at the shallower depth; so in principle the RMS difference at the shallower depth should be weighted more heavily in the modeling process. Moreover, the automodeling software is not capable of correlating the relationships between particular parameters at different field sizes. Indeed, there is no guarantee that beam parameters follow physically meaningful trends as functions of field size. The physicist cannot be confident that beam parameters obtained by interpolation provide a realistic description of the dose distribution. Consequently, the modeling methodology described in this paper utilizes the automodeling software to obtain initial values for some of the beam parameters. These parameters do not provide a sufficiently accurate beam model, so the parameters are then manually fine‐tuned as functions of field size.

#### Beam commissioning process

The vendor of the ${\text{PINNACLE}}^{3}$ treatment planning system provides “generic” beam models for the more common treatment machines. Once we selected a treatment machine for modeling, we used the appropriate generic model as a starting point for the modeling process. Doses calculated using the generic model, however, did not meet our accuracy criteria. Automodeling software that was provided with the treatment planning system was used to fit the measured data for all field sizes (4 cm × 4 cm to 40 cm × 40 cm) to a single set of beam parameters. Profiles calculated using the automodeled parameters were compared with the measured data. In all cases, differences between the calculated and the measured relative dose profiles were greater than the allowable tolerances. Fine‐tuning the automodeled parameters created field‐size‐specific beam models. Typically, models were constructed for 4 cm × 4 cm, 10 cm × 10 cm, 20 cm × 20 cm, and 40 cm × 40 cm fields. Interpolated models were then validated to fit the measured data for the intermediate sized fields. For some machines, especially at larger field sizes, the interpolated parameters failed to provide sufficiently accurate agreement between calculated and measured data. In such circumstances, beam models for intermediate field sizes were established with parameters determined to provide accurate agreement. In such cases, it was important to ascertain that the new beam parameters for the intermediate field size was logically consistent with parameters for other field sizes. It was also important to recheck the interpolated beam parameters for other intermediate field sizes, if data for such additional intermediate sizes exist. Models were first constructed for the open fields, then copied to the wedged fields and modified to fit the measured wedged field data.

The first beam parameters to be manually adjusted were the spectral weights. The energy bins at which the spectral weights were specified were the same as provided by the “generic” beam model. Electron contamination was turned off so that only the incident photon spectrum contributed to the central‐axis depth dose. Only the two or three highest energy spectral components needed to be adjusted to fit the calculated relative depth doses to the measured values. Weights were adjusted so that the differences between the calculation and measurement for depths beyond $d_{\max}$ were within specified tolerances. Depths slightly beyond $d_{\max}$ are depths at which the electron contamination also contributed to the central‐axis dose. In the absence of the electron contamination, the calculated doses at these shallow depths were often as much as 2% less than the measured values. We reduced this dose difference by softening the beams as much as feasible without compromising doses at large depths. Improving the fit at shallow depths minimized the contribution from electron contamination, making this contribution a correction to the central‐axis depth dose rather than a major component of the central‐axis depth dose at shallow depths. The goals in fitting the spectral weights were thus as follows: (1) make the dose differences at shallow depths beyond $d_{\max}$ small and negative, typically within 1%, and (2) make the dose differences beyond $d_{m}$, the maximum depth of electron contamination (as set in the generic beam model), within 0.5% for shallow depths (less than 20 cm) and 1.0% for large depths (greater than 20 cm). Tolerances were occasionally relaxed for larger depths to ensure tighter tolerances at shallower depths, which are considered more clinically significant.

Spectral weights were determined for the largest field size (40 cm × 40 cm) first, then those for the other field sizes. Care was taken to ensure that spectral weights changed with field size in a smoothly varying and physically meaningful manner. The spectral weights for the highest energy components should increase with decreasing field size, corresponding to the empirical observation that smaller beams are harder. Models for the 4 cm × 4 cm, 10 cm × 10 cm, 20 cm × 20 cm, and 40 cm × 40 cm field sizes were determined first. Then, the central‐axis depth doses for all measured intermediate field sizes were calculated and compared with the measured data to verify adequate agreement. If agreement was not satisfactory, spectral weights for the modeled field sizes were adjusted to bring the dose differences within tolerance. Occasionally it was not possible to bring the dose differences for intermediate sized fields within tolerance because of the inadequacy of linear interpolation of parameters between the modeled field sizes. In those cases, we generated additional models for these intermediate field sizes to bring calculated profiles into agreement with measurement. This occurred most often for the larger field size models (25 cm × 25 cm or 30 cm × 30 cm).

The next set of parameters to be determined were those that modeled the off‐axis dose profiles. These parameters were determined by fitting profiles for the largest field‐size beam (40 cm × 40 cm). First, the cone radius and the cone angle were computed for the largest field size beam using automodeling. The automodeling algorithm provided by the vendor omitted incorporation of the profile at $d_{\max}$. Consequently, manual fitting was required, including fitting to the profile at $d_{\max}$. The differences in relative doses were displayed as percentages of the dose on the central axis. Thus at greater depths a larger difference equated to a small percent difference when expressed in terms of either dose at $d_{\max}$ or dose at a typical prescription depth. The cone angle was modified first to fit the off‐axis profiles near the central axis. Then the cone radius was modified to fit the profiles at large distances from the central axis. Agreement between the calculated and measured off‐axis profiles within 2% of the dose at the central axis was usually achieved. Occasionally, greater discrepancies were accepted at the largest depth (typically 35 cm) to ensure a better fit at the shallower depths.

The source size was manually adjusted until the calculated profile slope in the penumbra region matched the measured slope. Because the present version of the treatment planning system does not have quantitative tools for this assessment, this match had to be estimated visually. In some cases, the calculated high‐gradient regions did not coincide with the measured high‐gradient regions. This was due to two reasons. If the actual width of the measured radiation field were not equal to the nominal width, the high‐gradient regions were displaced symmetrically on both sides of the radiation field. This was corrected by labeling the measured beam profiles with the correct field size, e.g., changing the collimator identifications for a 10 cm × 10 cm field to those for a 10.2 cm×10.2 cm field, resulting in calculated beam profiles that were 0.2‐cm wider at isocenter. A second source of disagreement of high‐gradient regions was due to a displacement in the measurement process. In this case, displacement was in the same direction on both sides of the radiation field. Tools in the data transfer software in the treatment planning system were used to displace the measured dose profiles and achieve a better fit. Displacement of the measured dose distributions was rare.

The three out‐of‐field profile parameters were fine‐tuned. Although the most obvious effects of these parameters occur in the shape of the off‐axis profile in the out‐of‐field region, these parameters can greatly influence the absolute dose delivered to the central axis, as shown in the results section of this paper. In order to minimize the influence on the dose delivered to the central axis, the Gaussian height parameter should be as small as possible. We first set the Gaussian height parameter to 0. Then the jaw transmission parameter was set to optimize the agreement between the calculation and measurement for the points on the off‐axis profiles that were most distant from central axis. Finally, the Gaussian height parameter was increased slightly (to a value typically between 0.01 and 0.05) to improve the fit between the slopes of the calculated profiles and the slopes of the measured profiles in the out‐of‐field region.

The profile parameters for the largest field size (40 cm × 40 cm) were copied to the models for the smaller fields (4 cm × 4 cm, 10 cm × 10 cm, and 20 cm × 20 cm). It was occasionally necessary to fine‐tune these smaller field models to achieve the agreement goals. Central axis depth doses and off‐axis profiles were calculated for field sizes intermediate to the modeled sizes to ensure that the interpolated models reproduced the measured profiles to within the specified accuracy criteria.

The last step was to model the electron contamination. The automodeling software was used to obtain the initial values of parameters. These values were then manually fine‐tuned. When possible, the depth fraction *DF* was set to 0 and the surface dose factor *SF* was set to 1 because it was usually unnecessary to model the electron contamination curve with a linear segment. For depths less than $d_{\max}$, i.e., in the build‐up region, we allowed disagreement between the calculated and measured central‐axis depth doses to be significantly larger than that allowed beyond the build‐up region. The Task Group 53 report^1^ suggests a goal of agreement within 20%; we found that agreement within 5% was readily achievable. More important than that agreement, the value of $d_{\max}$ must be accurately represented in the beam model. The electron contamination parameters were first fit for the largest field size, then for the smallest field size, and finally for the intermediate field sizes. Parameters were changed as little as possible among field sizes, and the percentage of difference between the calculated and measured central‐axis profiles was kept within 5% in the build‐up region and within 0.5% from $d_{\max}$ to $d_{m}$, the maximum depth of electron contamination. Central‐axis depth doses for the intermediate field sizes were calculated and compared to the measured values.

Parameters for the wedged‐field models were copied from the open‐field models, and then fine‐tuned to match the measured relative dose distributions. Parameters did not have to be modified substantially; the greatest modification was in the spectral weights to account for changes caused by the presence of the wedges. In addition, the beam modifier scatter parameter was adjusted to improve the fit of the off‐axis profiles. Further fine tuning of the calculated wedged‐field profiles was achieved by small modifications in the description of the physical profile of the wedge. Small changes of less than 1 mm in thickness resulted in a several percent change in the calculated beam profile. Such changes can be easily implemented for a single energy machine. For a dual energy machine the physical shape of the wedge affects profiles for both energies to different degrees. In a few cases we could not achieve satisfactory results with a single physical wedge profile for both energies, so we created separate machines for each of the two photon energies. We could then individually alter the wedge shape to obtain better results.

Table I summarizes the beam parameters and their effects on the calculated beam profiles.

**Table I:** Summary of beam parameters and their effects on calculated beam profiles.

Parameter	Effect	Magnitude of effect
**Energy Spectral Weight:**	An increase in the spectral weights of the higher energy components increases the central‐axis depth dose at larger depths and decreases the depth dose at shallower depths.	Changing the spectral weight of the highest energy component by as little as 0.001 will have a noticeable effect on the central‐axis depth dose. Different values of spectral weights for the two or three highest energy components are likely to be required for each field size.
**Incident Fluence**		
Fluence increase/unit distance (cone angle)	An increase in this parameter increases the magnitude of the off‐axis profile at all distances from the central axis.	Changing this parameter by 0.005 will have a noticeable effect on the off‐axis ratio, especially at larger distances from the central axis. The value of this parameter is likely to be insensitive to field size.
Fluence cone radius	An increase in the cone radius decreases the magnitude of the off‐axis profile at large distances from the central axis.	The off‐axis ratio is relatively insensitive to the value of this parameter. The cone radius is not a meaningful parameter for small field sizes.
Source size *X* Source size *Y*	An increase in the source size decreases the slope of the off‐axis profile in the high‐dose gradient region.	Typically a change of 0.1 is required to have a noticeable effect on the slope of the penumbra. The values of these parameters are likely to be insensitive to field size.
Gaussian height	An increase in the value of this parameter decreases the sharpness of the transition between the low‐dose gradient region outside the radiation field and the high‐dose gradient region.	The accuracy of monitor unit calculations for elongated fields decreases with an increase in the value of this parameter of around 0.05. The value of this parameter increases with field size.
Gaussian width	This parameter has a similar effect on the off‐axis profile as the Gaussian height parameter.	Because of the low value of the Gaussian height parameter required for accurate monitor unit calculations, this parameter has little effect on off‐axis profile.
Jaw transmission	An increase in the value of this parameter increases the value of the off‐axis profile in the low‐dose gradient region outside the radiation field.	Changes in this parameter of 0.005 have a noticeable effect on the off‐axis profile in the low‐dose gradient region outside the radiation field. The value of this parameter typically increases with field size.
**Modifiers**		
Modifier Scatter Factor	This parameter affects off‐axis profiles for wedged fields. An increase in the value of this parameter decreases the value of the off‐axis ratio in the region of the thin end of the wedge and increases the off‐axis ratio in the region of the thick end of the wedge.	Changes in this parameter of 0.1 are needed to have a noticeable effect on the off‐axis profile. The value of this parameter typically increases with field size.
**Electron Contamination**		
Maximum Depth (cm)	The value of this parameter is determined by the energy of the radiation beam and is typically set to be approximately $2\times d_{\max}$.	The exact value of this parameter is not too critical. It is independent of field size.
Surface dose (Dose/Fluence)	Increasing the value of this parameter increases the dose at very shallow depths corresponding to the region of electron contamination.	The exact value of this parameter is not critical, as the tolerance of the accuracy of the model near the surface is significantly looser than elsewhere.
Depth coefficient (1/cm) (*K*)	Increasing the value of this parameter decreases the amount of electron contamination at depths beyond the surface.	Changes in this parameter of 0.5 are needed to have a noticeable effect on the central‐axis depth dose at shallow depths.
Off‐axis coefficient $\left(1/{\text{rad}}^{2}\right)$ (*A*)	Increasing this parameter makes the electron contamination component more forward peaked.	Because we have not seen any significant effect of this parameter on the dose distribution we set its value to 0.
Depth fraction (*DF*)	Increasing the value of this parameter extends the linear portion of the electron contamination to greater depths.	Because we do not use the linear portion of the electron contamination curve for modeling we set this parameter value to 0.
*SF*	Increasing the value of this parameter decreases the magnitude of the electron contamination curve in the exponential region.	Because we do not use the linear portion of the electron contamination curve for modeling we set this parameter value to 1.
$C_{1}$, $C_{2}$, $C_{3}$	These three parameters affect the field size dependence of the electron contamination.	We use a single set of values determined by the automodeling.
**Spectral factors**		
Off‐axis Softening Factor (S)	Increasing the off‐axis softening parameter increases the value of the off‐axis profile at large distances from the central axis for shallow depths.	Changes in the value of this parameter of 0.01 are observable in the off‐axis profiles. The value of this parameter is typically independent of field size.
**Modeling geometry**		
Fluence grid resolution, Phantom size—lateral Phantom size—depth	The values of these parameters are determined by the geometry of the beam.	

Calculations of monitor units were checked against independent calculations for a variety of treatment conditions. These calculations can reveal very subtle errors in beam models that cannot be detected by looking at relative dose distributions.^13^ We tested approximately 150 configurations of different field sizes, depths, wedges, and blocking for monitor unit verification. In order to overcome a systematic discrepancy of approximately 0.5%, it was necessary to increase the measured machine output for a 10 cm × 10 cm field at 10‐cm depth so that the calculated monitor units accurately reproduced measurement for this field size.

The results of beam modeling were extensively documented. All profiles were printed. We summarized the results by recording the greatest discrepancies between the calculated and measured profiles for several regions of interest. We also calculated and documented the dose distributions for each field size for which we had measured data. Doses were calculated in a water phantom, and beams were weighted to permit a direct comparison with the measured dose distributions. Because beam modeling matched the calculated central‐axis dose profiles to the measured central‐axis doses at 10‐cm depth, beams were weighted so that they delivered a dose at 10‐cm depth equal to the measured percent depth dose at that depth. We superimposed transparency hard copy plots of the measured dose distributions on hard copy isodose plots of the calculated‐dose distributions.

### Open beams

To date, we have commissioned 10 photon beam models from seven different therapy machines in our clinic at M.D. Anderson: five Clinac 2100‐C linear accelerators (Varian Oncology Systems, Palo Alto, CA) with three different sets of beam characteristics, a Cobalt‐60 teletherapy unit (Theratronics International Ltd., Kanata Ontario, Canada), two Clinac 600‐C accelerators (Varian Oncology Systems, Palo Alto, CA) with matched beam characteristics, a Mevatron 6740 linear accelerator (Siemens Medical Systems, Concord, CA) and a Mevatron K‐80 linear accelerator (Siemens Medical Systems, Concord, CA). A 6‐MV and an 18‐MV photon beam model has been commissioned for each of the Clinac 2100‐C accelerators, 6‐MV photon beam models have been commissioned for the Clinac 600‐C and Mevatron 6740 accelerators, and an 18‐MV photon beam model has been commissioned for the Mevatron K‐80. We present the results of our beam commissioning for the 6‐MV photon beam model on a Mevatron 6740 linear accelerator; comparable results were obtained for the other machines and beams.

Parameters for the generic Mevatron 6740 6‐MV photon beam model provided by the vendor of the treatment planning system are listed in Table II. In general, this model provided relatively good agreement with the measured profiles. At depths between $d_{\max}$ and 2.5 cm, the maximum depth of electron contamination, the calculated central‐axis profiles were within 1% of the measurement. For large field sizes (40 cm × 40 cm) they were within 2% of measurement. At depths beyond 2.5 cm, the calculated central‐axis profiles were within 1% of the measured values for all field sizes. In the high‐dose, low‐gradient region, the values of the calculated off‐axis profiles were less than those of the measured beam, indicating a need to increase the cone angle. In the low‐dose, low‐gradient region, the calculated profile values were up to 3% greater than the measured profile values, especially at shallow depths. In the high‐gradient region, the penumbra appeared too wide, indicating a need to decrease the source size. The high precision that is indicated in these manufacturer‐supplied parameter values is, for the most part, unnecessary. Small changes in most of the beam parameters have little, if any, effect on the calculated profiles. Moreover, the modeling geometry using a phantom width of 50 cm is not adequate for large fields such as the 40 cm × 40 cm field.

**Table II:** Beam parameters for a Mevatron 6740 6‐MV photon beam.

Energy spectrum	Generic Automodeled	
Energy (MeV)	Relative photon weights	
0.10	0.059	0.057
0.20	0.102	0.100
0.30	0.139	0.137
0.40	0.172	0.169
0.50	0.199	0.196
0.60	0.223	0.219
0.80	0.257	0.253
1.00	0.279	0.273
1.25	0.290	0.283
1.50	0.289	0.281
2.00	0.260	0.251
3.00	0.165	0.157
4.00	0.082	0.076
5.00	0.032	0.028
6.00	0.010	0.007
8.00	0.000	0.000
**Incident fluence**		
Incident fluence increase/cm	0.00852307	0.0115108
Incident fluence cone radius (cm)	13.3592	14.587
*X* (perpendicular to gantry axis) (cm)	0.1025	0.23875
*Y* (parallel to gantry axis) (cm)	0.11375	0.07625
Gaussian height (cm)	0.0790897	0.089879
Gaussian width (cm)	0.824079	0.895998
Jaw transmission	0.0100608	0.00673947
**Modifiers**		
Modifier scatter factor	0.2	0.2
**Electron contamination**		
Maximum depth (cm)	2.5	2.5
Surface dose (dose/fluence)	0.0005	0.0005
Depth coefficient (1/cm)	0.3	0.3
Off‐axis coefficient $\left(1/{\text{rad}}^{2}\right)$	0	0
*DF*	0	0
*SF*	1	1
$C_{1}$ (dose/fluence)	0.00193502	0.00193502
$C_{2}$ (dose/fluence)	0.0661798	0.0661798
$C_{3}\;(1/\text{cm})$	0.00375271	0.00375271
**Spectral factors**		
Off‐axis softening factor	4.53797	0.140592
**Modeling geometry**		
Fluence grid resolution (cm)	0.40	0.40
Phantom size–lateral (cm)	50.00	50.00
Phantom size–depth (cm)	50.00	50.00

Automodeling all beam parameters with a single set of values based on all measured profiles produced the parameters also listed in Table II. Agreement with measured profiles improved slightly over the generic parameters. However, field‐size specific profiles are necessary to achieve clinically acceptable agreement.

Separate beam models were then produced for 4 cm × 4 cm, 10 cm × 10 cm, 20 cm × 20 cm, and 40 cm × 40 cm open fields as described in the beam‐commissioning methodology section (Table III). The parameters for these beam models are far less precise than those obtained with either the generic model or automodeling. However, these parameters more accurately represent the measured beam profiles. Furthermore, the field‐size variations are rather small and consistent among field sizes; thus it is anticipated that the interpolated models for the intermediate field sizes should accurately reproduce the measured data.

**Table III:** Field‐size specific beam parameters for open fields.

**Energy spectrum**	4×4	10×10	20×20	30×30	40×40
	field	field	field	field	field
Energy (MeV)	Relative photon weights			
0.10	0.057	0.057	0.057	0.057	0.057
0.20	0.100	0.100	0.100	0.100	0.100
0.30	0.137	0.137	0.137	0.137	0.137
0.40	0.169	0.169	0.169	0.169	0.169
0.50	0.196	0.196	0.196	0.196	0.196
0.60	0.219	0.219	0.219	0.219	0.219
0.80	0.253	0.253	0.253	0.253	0.253
1.00	0.273	0.273	0.273	0.273	0.273
1.25	0.283	0.283	0.283	0.283	0.283
1.50	0.281	0.281	0.281	0.281	0.281
2.00	0.251	0.251	0.251	0.251	0.251
3.00	0.157	0.157	0.157	0.157	0.157
4.00	0.076	0.076	0.076	0.076	0.076
5.00	0.030	0.025	0.025	0.025	0.025
6.00	0.005	0.005	0.005	0.005	0.005
8.00	0.000	0.000	0.000	0.000	0.000
**Incidence fluence**					
Incident fluence increase/cm	0.016	0.016	0.016	0.013	0.011
Incidence fluence cone radius (cm)	3	3	5	9	15
*X* (perpendicular to gantry axis) (cm)	0.1	0.1	0.1	0.1	0.1
*Y* (parallel to gantry axis) (cm)	0.1	0.1	0.1	0.1	0.1
Gaussian height (cm)	0.03	0.03	0.03	0.03	0.03
Gaussian width (cm)	1.3	1.3	1.3	1.3	1.3
Jaw transmission	0.01	0.02	0.03	0.04	0.05
**Modifiers**					
Modifier scatter factor	0.2	0.2	0.2	0.2	0.2
**Electron contamination**					
Maximum depth (cm)	2.5	2.5	2.5	2.5	2.5
Surface dose (dose/fluence)	0.22	0.22	0.22	0.22	0.22
Depth coefficient (1/cm)	3.2	3.2	3.2	3.2	3.2
Off‐axis coefficient $\left(1/{\text{rad}}^{2}\right)$	0	0	0	0	0
*DF*	0	0	0	0	0
*SF*	1	1	1	1	1
$C_{1}$ (dose/fluence)	0.015	0.015	0.015	0.015	0.015
$C_{2}$ (dose/fluence)	0.07	0.07	0.07	0.07	0.07
$C_{3}\;(1/\text{cm})$	0.07	0.07	0.07	0.07	0.07
**Spectral factors**					
Off‐axis softening factor	0	0	0	0	0
**Modeling geometry**					
Fluence grid resolution (cm)	0.40	0.40	0.40	0.40	0.40
Phantom size—lateral (cm)	50.00	50.00	50.00	60.00	70.00
Phantom size—depth (cm)	50.00	50.00	50.00	50.00	50.00

For some machines, however, the interpolated models for the intermediate field sizes did not provide us with sufficiently accurate reproductions of the measured dose values. This was especially true for the larger field sizes. In cases in which the interpolated model did not reproduce the measured dose values with sufficient accuracy, we created a new set of parameters for the intermediate field size that generated a more accurate beam model. As seen from Table III, a separate model had to be created for the 30 cm×30 cm field for the Mevatron 6740.

**Figure 2:**
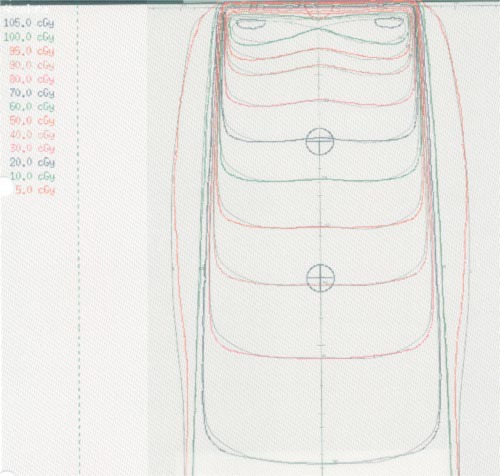
(Color) Isodose distributions for a 14 cm × 14 cm open field. The calculated dose distribution is indicated in color, and the measured dose distribution is indicated in black and white.

Calculated profiles were compared with the measured profiles for all field sizes for which measurements were available (Table IV). Because the beams were manually modeled to preferentially fit the measured off‐axis profiles at shallower depths, the discrepancy between the calculated and measured off‐axis profiles is often large for the profile measured at the maximum depth of 35‐cm. However, these differences are expressed as percentages of the dose along the central axis at depth, so that a large percentage difference at a 35‐cm depth actually corresponds to a relatively small absolute‐dose difference. Thus Table IV displays the largest percentage discrepancy between the calculation and measurement at a 35‐cm depth separately from the largest discrepancy at shallower depths. In Table IV the description “inside beam” refers to points inside the beam penumbra for which the dose gradient is small. These points were determined by inspection of the calculated profile dose values, and generally included points for which the off‐axis profile was greater than 0.95. The description “outside beam” refers to points outside the beam penumbra for which the dose gradient is small. These points, as well, were determined by inspection of the calculated profile dose values. An example of the comparison of dose distributions is illustrated in Fig. 2, which illustrates calculated and measured dose distributions for a 14 cm × 14 cm open field. The original dose comparisons are obtained by placing a transparency of the measured dose distribution over a hardcopy of the calculated dose distribution. These figures are representative of all isodose distribution comparisons for this machine.

**Table IV:** Maximum deviation between calculated and measured dose profiles. “Inside beam” refers to points inside the beam penumbra for which the dose gradient is small, while “outside beam” refers to points outside the penumbra for which the dose gradient is small.

			Central axis		Off‐axis inside beam		Off‐axis outside beam		
Field size (cm)	$d_{\max}$ (cm)	$\left(1.0\;\text{cm}-d_{\max}\right)$	$\left(d_{\max}-19\;\text{cm}\right)$	(19−40 cm)	<35 cm	35 cm	<35 cm	35 cm	Penumbra (>20%)
4×4	1.80	2.5%	0.2%	0.3%	0.5%	0.3%	1.1%	1.3%	<1 mm
5×5	1.60	2.1%	0.2%	0.3%	1.0%	0.2%	1.4%	2.1%	<1 mm
6×6	1.60	1.7%	0.4%	0.3%	1.2%	0.7%	1.8%	2.2%	1 mm
8×8	1.60	1.5%	0.3%	0.2%	1.0%	1.1%	1.3%	1.9%	<1 mm
10×10	1.60	1.4%	0.3%	0.2%	1.0%	1.6%	1.5%	2.0%	1 mm
12×12	1.60	1.5%	0.3%	0.2%	1.2%	1.5%	2.0%	1.9%	1 mm
14×14	1.60	1.3%	0.3%	0.2%	1.0%	1.2%	1.7%	2.7%	1 mm
16×16	1.60	0.7%	0.3%	0.1%	1.0%	1.4%	1.6%	2.5%	1 mm
18×18	1.50	0.5%	0.3%	0.2%	1.2%	1.3%	2.2%	3.0%	1 mm
20×20	1.50	1.0%	0.5%	0.2%	0.8%	1.4%	1.8%	3.0%	1 mm
25×25	1.45	0.7%	0.3%	0.2%	1.9%	3.1%	2.5%	3.7%	2 mm
30×30	1.40	0.5%	0.6%	0.2%	2.5%	3.9%	2.8%	4.6%	2 mm
40×40	1.30	0.7%	1.0%	0.3%	3.2%	4.5%	3.2%	5.6%	3 mm

### Wedged beams

We found that when using the open‐field models to calculate the wedged‐field dose distributions, we needed to make only minor changes in the beam model to fit the wedged‐field data accurately. Parameters for the 60° wedged field, the most difficult to model, are shown in Table V.

**Table V:** Beam parameters for 60° wedged fields.

	4×4	10×10	15×15
Energy spectrum	field	field	field
Energy (MeV)		Relative photon weights	
0.10	0.057	0.057	0.057
0.20	0.100	0.100	0.100
0.30	0.137	0.137	0.137
0.40	0.169	0.169	0.169
0.50	0.196	0.196	0.196
0.60	0.219	0.219	0.219
0.80	0.253	0.253	0.253
1.00	0.273	0.273	0.273
1.25	0.283	0.283	0.283
1.50	0.281	0.281	0.281
2.00	0.251	0.251	0.251
3.00	0.157	0.157	0.157
4.00	0.076	0.076	0.076
5.00	0.025	0.008	0.008
6.00	0.005	0.000	0.000
8.00	0.000	0.000	0.000
**Incident fluence**			
Incident fluence increase/cm	0.012	0.012	0.014
Incident fluence cone radius (cm)	3	3	4
*X* (perpendicular to gantry axis) (cm)	0.1	0.1	0.1
*Y* (parallel to gantry axis) (cm)	0.1	0.1	0.1
Gaussian height (cm)	0.03	0.03	0.03
Gaussian width (cm)	1.3	1.3	1.3
Jaw transmission	0.02	0.03	0.05
**Modifiers**			
Modifier scatter factor	0.12	0.12	0.20
**Electron contamination**			
Maximum depth (cm)	2.5	2.5	2.5
Surface dose (dose/fluence)	1.0	1.0	1.0
Depth coefficient (1/cm)	9.9	9.9	9.9
Off‐axis coefficient $\left(1/{\text{rad}}^{2}\right)$	0	0	0
*DF*	0	0	0
*SF*	1	1	1
$C_{1}$ (dose/fluence)	0.05	0.05	0.05
$C_{2}$ (dose/fluence)	0.8	0.8	0.8
$C_{3}\;(1/\text{cm})$	0.1	0.1	0.1
**Spectral factors**			
Off‐axis softening factor	0.004	0.004	0.004
**Modeling geometry**			
Fluence grid resolution (cm)	0.40	0.40	0.40
Phantom size—lateral (cm)	50.00	50.00	50.00
Phantom size—depth (cm)	50.00	50.00	50.00

In general, we needed to soften the beam spectrum slightly by decreasing the spectral weights of the highest energy components. This is contrary to the observation that a polyenergetic beam passing through a wedge should be hardened. We believe that although beam hardening caused by the transmission of the beam through the wedge may be modeled appropriately, the model does not account for scatter from the wedge. Consequently, the open‐field model is somewhat harder than the actual beam that is transmitted through the wedge. Thus we required a softer beam model for the wedged fields. The open‐field model also underestimated the radiation outside the field; this was accounted for in the wedged‐field model by slightly increasing the jaw transmission parameter.

With some wedged‐field models, especially those with a large wedge angle, we observed large discrepancies between the calculated and the measured off‐axis profiles near the thin end of the wedge. The calculated off‐axis doses in this region were often greater than the measured values for shallow depths, and smaller than the measured values for large depths. This implies that the beam is too soft near the thin end of the wedge. In theory, one could harden the beam at the periphery of the field by decreasing the off‐axis softening parameter. The behavior of the calculated off‐axis doses, however, occurred in some machines even when the off‐axis softening parameter had a zero value. Furthermore, the off‐axis softening parameter affects the beam profile symmetrically around the central axis. Because the depth dependence of the off‐axis profiles in the thick end of the wedge appeared to be correct, the spectrum in the thick end of the wedge did not have to be modified. The only parameter that affects the wedged‐field profiles asymmetrically is the modifier scatter parameter. Increasing the value of this parameter decreased the calculated dose near the thin end of the wedge and increased it near the thick end of the wedge. Unfortunately, this modification affects the beam profiles at all depths; we would need to modify the profile in different directions at different depths.

**Figure 3:**
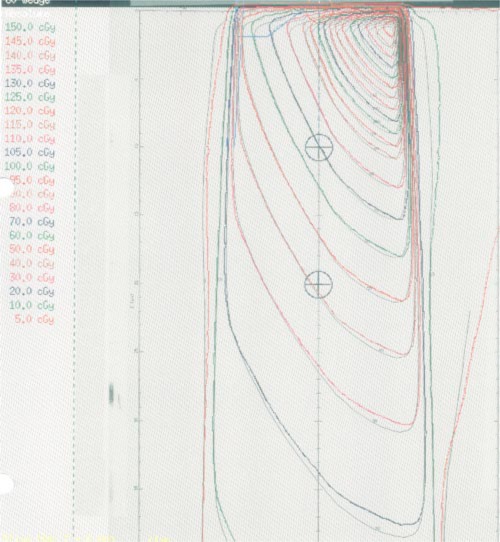
(Color) Isodose distributions for a 12 cm × 12 cm 60° wedged field. The calculated dose distribution is indicated in color and the measured dose distribution is indicated in black and white.

Comparisons between calculated and measured beam profiles are illustrated in Table VI, and a representative comparison of dose distributions for a 12 cm × 12 cm 60° wedged field is illustrated in Fig. 3.

**Table VI:** Maximum deviation between calculated and measured dose profiles for 60° wedged fields. Maximum difference at depth of 1.5 cm. Calculated value at other depths within 2.0% of measurement. Maximum difference at depth of 1.5 cm. Calculated value at other depths within 2.5% of measurement.

			Central axis		Off‐axis inside beam		Off‐axis outside beam		
Field Size (cm)	$d_{\max}$ (cm)	$\left(1.0\;\text{cm}-d_{\max}\right)$	$\left(d_{\max}-19\;\text{cm}\right)$	(19−40 cm)	<35 cm	35 cm	<35 cm	35 cm	Penumbra (>20%)
4×4	1.70	3.7%	0.4%	0.6%	1.9%	1.8%	0.9%	0.7%	<1 mm
5×5	1.70%	3.8%	0.5%	0.7%	2.9%a	2.3%	1.0%	1.6%	<1 mm
6×6	1.75	4.1%	0.5%	0.8%	2.5%a	2.1%	1.3%	1.8%	<1 mm
8×8	1.80	4.3%	0.3%	0.8%	3.2%b	2.4%	1.2%	1.7%	<1 mm
10×10	1.70	4.1%	0.6%	1.0%	3.2%	1.7%	1.3%	2.0%	1 mm
12×12	1.70	5.0%	0.5%	0.9%	1.6%	1.8%	2.4%	2.5%	1 mm
15×15	1.60	2.3%	0.4%	1.0%	2.5%	1.5%	3.4%	3.0%	2 mm

### Monitor units

Even after commissioning a large number of beams, we still had considerable difficulty making our monitor unit calculations match the monitor units obtained from straightforward point dose calculations using tissue‐maximum ratio (TMR) tables. We found that the monitor units calculated using the treatment planning system differed from those calculated using TMR tables by as much as 4–5 %. Moreover, we obtained substantially different values for monitor unit calculations for rectangular fields that depended on which beam edges the upper collimator jaws defined and which the lower jaws defined. Although some difference might be expected, the differences indicated by the treatment planning system were substantially greater than those obtained by the measurement. We found that the incorrect calculation of the monitor units was related to incorrect modeling of the low‐dose low‐gradient region using the Gaussian height and Gaussian width parameters. Only when the Gaussian height parameter was kept small were the monitor unit calculations correct.

A minor, but systematic, discrepancy between monitor units calculated using the treatment planning system and those calculated from independent TMR values resulted from the different methods of ray tracing done in the physics‐commissioning component of the treatment planning system compared to the patient‐planning component of the system. All our monitor unit calculations, made using a water phantom in the patient‐planning component of the system, initially indicated a systematic underestimation of dose that averaged approximately 0.5%. In the physics‐commissioning component of the system, depths are measured exactly from the surface of the calculation phantom. In the patient‐planning component of the system, ray tracing is done through a unit density phantom comprised of voxels. The centers of the voxels lying on the surface of the phantom define the phantom surface. Therefore, depths are calculated to be a half voxel thickness more than in the physics commissioning. For a beam with an SSD of 100 cm the ray along the central axis will intersect the first voxel at a distance of 100 cm minus half a voxel thickness. Points at depth are then calculated to lie at depths greater by half a voxel thickness, resulting in a discrepancy in the dose calculation. In order to account for this discrepancy, the measured machine output for a 10 cm×10 cm field at a 10 cm×10 cm depth was increased by an amount required to give the correct number of monitor units for a 10 cm×10 cm field. After this modification was made, the discrepancies between ${\text{PINNACLE}}^{3}$ calculated monitor units and TMR‐calculated monitor units for square fields averaged to zero.

## DISCUSSION

The goals of beam commissioning were achieved in all the beams we commissioned. Typical results are shown for the Mevatron 6740 6‐MV beam in Tables V and VI and Figs. 2 and 3. For this beam, in the build‐up region, the calculated relative doses generally lie within 2% of the measured doses. Beyond the buildup region, the calculated relative doses generally lie within 0.5% of measurement. The calculated off‐axis profiles generally lie within 2% of measurements.

Shortcomings in the beam simulation and algorithms led to parameter values that exhibited behavior different from that expected physically. For example, one would expect the energy spectrum for a wedged field to be somewhat harder than that for an unwedged field. Yet, because of the approximations in the beam simulation, it was necessary to model the energy spectrum for a wedged field with a softer beam than that for an unwedged field. Asymmetric changes in the off‐axis energy spectrum are not adequately simulated, leading to further discrepancies between the calculation and measurement. Inadequate simulation of extra‐focal radiation led to parameter values that accurately modeled the off‐axis profiles but that resulted in monitor‐unit calculations that were clinically unacceptable. Uncertainties in the precise location of the patient surface due to the finite size of the CT voxel led to small, but systematic, discrepancies in the calculation of the monitor units. Consequently, the process of beam commissioning requires a set of compromises incorporating the clinical judgment of the medical physicist and is not yet be adequately approximated by the automodeling scheme in the planning system.

The automodeling software needs to be made more “intelligent.” A danger in using the automodeling software is that one can take it to extreme, generating a different model for each field size. Beam modeling must take into account the need for the beam parameters to be physically relevant and, particularly, to progress smoothly and consistently from one field size to another. Because fitting parameters requires compromising the tightness of the fit, the physicist must make those compromises physically and clinically meaningful. For example, to improve the fit at clinically meaningful depths, one may relax the fit requirements at depths that may be substantially greater than those at which treatment will be delivered.

Additional software, either within the treatment planning system or in an external unit, could make the beam‐commissioning process more efficient. The treatment planning system displays comparisons between the calculated and measured dose profiles; the ability to extract this information onto a spreadsheet would be very desirable. A spreadsheet would allow the physicist to do more extensive data analysis and to generate better documentation comparing the calculated and measured dose profiles. At present, the source size is determined either through the automodeling software by comparing relative dose values in the high‐gradient (penumbra) region or manually by estimating the coincidence between the calculated and measured penumbrae. The ability to calculate the slope of the off‐axis profile in the penumbra or the distance between the 20% and 80% decrements might improve the determination of the source size. In addition, the measured beam widths incorporate experimental uncertainties. By actually determining the distance between the 50% decrements, the true field size of the measured fields could be more accurately determined. The ability to extract dose matrices from the treatment planning system would also improve the beam‐commissioning process. This would allow two‐dimensional numerical comparisons to measured data, such as dose‐difference and distance‐difference plots, which can provide a much more vivid comparison of two methods of determining dose.

When we first began beam modeling for this system, several weeks were required to generate satisfactory photon beam models. However, once the methodology described in this paper was established, we found that we could obtain a beam model in approximately 24 man hours. An additional 40 to 80 man hours was needed to generate and review the documentation. Thus if necessary, we could allocate a three‐week time period from the time the beam data was acquired for a new machine until the time the beam could be released for clinical use.

The general approach to beam commissioning described in this paper attempts to model the beam using as few parameters as possible and fine‐tunes the parameter values in a systematic manner so that the field size dependence of these values corresponds to the physical interpretation of these parameters. Beam commissioning, especially for a complex beam model used in 3D treatment planning systems, remains to some extent an art. However, the adoption of a commissioning procedure such as that illustrated in this paper may help generate consistent and meaningful sets of beam parameters within an acceptable time frame. While this methodology has been designed for the implementation of a specific treatment planning system, the general approach may be applied as well to other treatment planning systems.
